# Peritoneal Dissemination of Hepatoid Carcinoma of the Ovary Treated with Cytoreductive Surgery and Hyperthermic Intraoperative Intraperitoneal Chemotherapy

**DOI:** 10.1155/2013/283295

**Published:** 2013-06-10

**Authors:** Pedro Antonio Cascales Campos, Jose Gil Martinez, Amparo Torroba, Francisco Machado, Pascual Parrila Paricio

**Affiliations:** ^1^Peritoneal Carcinomatosis Unit, Department of Surgery, Hospital Clínico Universitario Virgen de la Arrixaca, Carretera Madrid-Cartagena, El palmar, 30120 Murcia, Spain; ^2^Department of Pathology, Virgen de la Arrixaca University Hospital, Carretera Madrid-Cartagena, El palmar, 30120 Murcia, Spain; ^3^Department of Gynecological Oncology, Virgen de la Arrixaca University Hospital, Carretera Madrid-Cartagena, El palmar, 30120 Murcia, Spain

## Abstract

Hepatoid carcinoma of the ovary (HCO) was first reported in 1987 in 5 cases of malignant ovarian tumors which were similar to hepatocarcinoma in the histological analysis. We report the first case in the literature of a patient diagnosed with HCO treated using HIPEC after extensive cytoreductive surgery, and we discuss the value of this therapeutical option in patients with HCO.

## 1. Introduction

Hepatoid carcinoma of the ovary (HCO) was first reported in 1987 by Ishikura and Scully [1], as a malignant neoplasia of the ovary with microscopic architectural and cytological characteristics similar to well-differentiated hepatocarcinoma. Although some authors classify HCO within the group of malignant epithelial neoplasms of the ovary [2], currently there are some controversies about the real histological origin of the tumor [3]. The recent application of more aggressive therapies in peritoneal carcinomatosis of ovarian origin, through the administration of hyperthermic intraoperative intraperitoneal chemotherapy (HIPEC) after maximal effort cytoreduction, has provided a new way to improve results in the treatment of ovarian carcinoma with peritoneal carcinomatosis [4] and could even be useful in uncommon histologies such as HCO. We report the first case in the literature of a patient diagnosed with HCO treated using HIPEC after extensive cytoreductive surgery, and we discuss the value of this therapeutical option in patients with HCO.

## 2. Case Report

**Figure 1:**
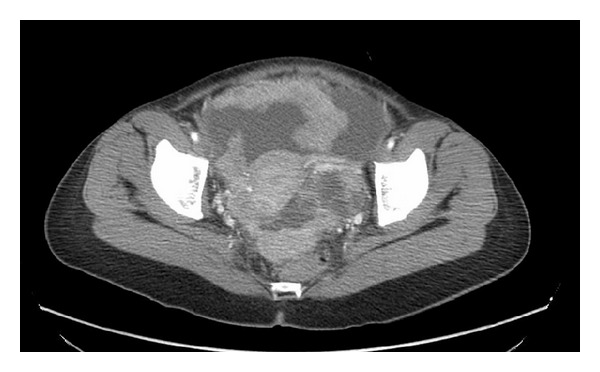
CT scan. Pelvic mass with ascites, peritoneal deposits, and enlargement of the greater omentum.

**Figure 2:**
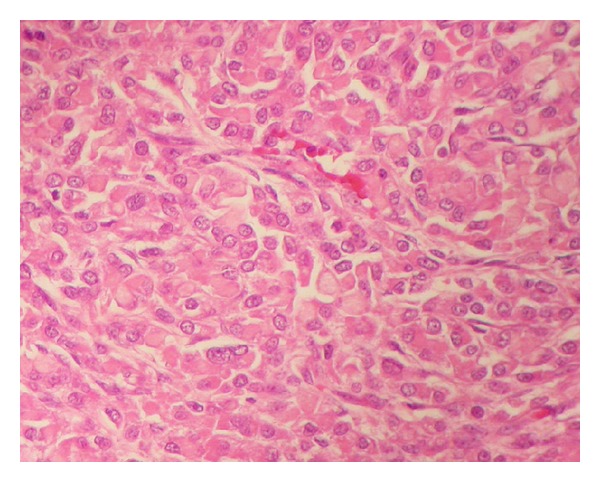
Columnar neoplasia made up of polygonal cells of eosinophilic cytoplasms, forming structures which look like the histological image that appears in well-differentiated hepatocarcinoma.

A 57-year-old female with no relevant medical history was referred to our unit with clinical symptoms consisting in abdominal discomfort and distension, nauseas, vomiting, and weight loss. On physical exploration, there was a palpable mass in the hypogastrium with high volume of ascites. Laboratory tests showed a high serum AFP level (397.0 ng/mL, normal, 0–5) and high serum CA125 level (1247 U/mL, normal, 0–35). A subsequent pelvic and abdominal computerized tomography (CT) scan demonstrated the presence of a large pelvic mass, ascites, and omental cake, suggesting ovarian neoplasia with peritoneal dissemination (IIIC-stage) (Figure 1). One gastroscopy and colonoscopy were normal. After xifo-pubic laparotomy, surgical exploration was performed, finding a large pelvic mass of 12 × 12 × 12 cms which infiltrated the uterus and pelvic peritoneum, as well as implants at the Douglas pouch, massive infiltration of the greater omentum, peritoneal implants in the right hemidiaphragm, and abundant ascites (4-5 liters). The peritoneal carcinomatosis index (PCI) was calculated, according to Sugarbaker's criteria (PCI = 9). A complete cytoreduction surgery included pelvic peritonectomy with total hysterectomy and bilateral salpingo-oophorectomy including Douglas pouch, complete omentectomy, right diaphragmatic peritonectomy, and appendectomy. After complete cytoreduction (no gross visible tumor), hyperthermic intraoperative intraperitoneal chemotherapy was used at 42°C during 60 minutes using paclitaxel (dose of 60 mg/m^2^). The patient was discharged on the sixth postoperative day without any adverse event. The histological analysis revealed the presence of hepatoid ovarian carcinoma (Figure 2) with both reactive interstitial proliferation and images of vascular invasion. Adjuvant treatment was completed with a combination of 6 cycles of systemic chemotherapy with carboplatin (AUC × 6) and paclitaxel (175 mg/m^2^). Currently, she has metastasis in the L2 vertebra treated with radiotherapy, without any signs of peritoneal relapse, 28 months after surgery.

## 3. Discussion 

HCO was first reported by Ishikura and Scully in 1987, after discovering 5 cases of malignant ovarian tumors which were similar to hepatocarcinoma in the histological analysis [1]. What is more and in the same way as with hepatocarcinoma, the HOC showed positive immunohistochemical staining for the alpha-fetoprotein marker, which is often elevated in peripheral blood samples [5]. The positivity for alphaphetoprotein was initially considered by Ishikura as a determining factor for establishing a clear diagnosis of HCO and usually a characteristic feature of this type of tumors. 

In the histological analysis of this type of tumors, diffuse infiltration of the ovarian parenchyma is usually found due to a columnar neoplasia composed of polygonal cells with eosinophilic cytoplasms, forming structures which look like the histological image that appears in well-differentiated hepatocarcinoma [5]. In immunohistochemical staining, the cells can be positive for cytokeratin 7, AFP (alpha-fetoprotein), CD10 in a canalicular pattern and polyclonal CEA. They can also be positive for progesterone receptors [5]. Staining with Hep-Par 1, a very specific marker in mature hepatic cells is always negative [6].

In the genital area, 75% of cases were reported in the ovary. The absence of lesions in the liver parenchyma, upon diagnosis or during followup, would support the diagnosis of the primary ovarian origin of the neoplasia. The simultaneous presence of an ovarian and hepatic lesion at the time of diagnosis makes it very difficult to distinguish between primary ovarian or hepatic origin, although ovarian metastases of hepatocarcinoma are very uncommon [7]. The prognosis of the HCO is determined by its tendency to systemic dissemination, in which lung and bone metastases are common. In our case, observable vascular invasion already existed in the specimens provided for histological analysis and the patient was diagnosed with single vertebral metastasis 24 months after surgery.

The precise origin of the tumor cell is controversial. HCO has sometimes been classified within the group of primary ovarian tumors, in the “miscellaneous” section. The association of this carcinoma with areas of papillary serous carcinoma or areas of adenocarcinoma, in addition to its elevated Ca.125 marker, lends support to its epithelial origin [5]. However, the HCO should be distinguished too from other ovarian tumors, with nonepithelial origin, whose histology can also appear with hepatoid morphology as yolk sac tumours [8].

The therapeutic protocol published by Sugarbaker, initially for gastrointestinal tumors with peritoneal dissemination, proposes maximal surgical cytoreduction, which would eliminate macroscopic disease, accompanied by the administration of hyperthermic intraoperative intraperitoneal chemotherapy (with platins and taxanes in the case of the ovary) which would eradicate microscopic disease, the cause of relapses detected during followup [9]. Ideal candidates for this treatment would be tumors which have a tendency to intraperitoneal dissemination, with long periods of time in which the disease remains limited to the peritoneum cavity and neoplasias which are sensitive to chemotherapy. Epithelial ovarian carcinoma in general and HOC in particular are ideal cytoreduction and hyperthermic intraoperative intraperitoneal chemotherapy. Four of the five cases of HCO described in the original series by Ishikura and in more than 70% of those described in the literature involve disseminated peritoneal disease at diagnosis or in the followup. Due to the good results reported in the literature with HIPEC in ovarian cancer with peritoneal dissemination, there is currently a promising therapeutically option, although this is still not a standardized procedure for peritoneal carcinomatosis of ovarian origin [10]. 

To conclude, the use of peritonectomy procedures and the use of hyperthermic intraoperative intraperitoneal chemotherapy (HIPEC) are a therapeutic weapon which has demonstrated its utility in peritoneal dissemination in gastrointestinal and gynecological tumors. Ovarian carcinoma has been postulated as a firm candidate for this therapy and hepatoid carcinoma of the ovary could also benefit from this.
